# The Impact of Inflow Angle on Aneurysm Hemodynamics: A Simulation Study Based on Patient-Specific Intracranial Aneurysm Models

**DOI:** 10.3389/fneur.2020.534096

**Published:** 2020-12-23

**Authors:** Xiao Mo, Qianqian Meng, Xinjian Yang, Haiyun Li

**Affiliations:** ^1^Beijing Key Laboratory of Fundamental Research on Biomechanics in Clinical Application, School of Biomedical Engineering, Capital Medical University, Beijing, China; ^2^Beijing Neurosurgical Institute, Beijing Tiantan Hospital, Beijing, China

**Keywords:** intracranial aneurysms, hemodynamics, fully coupled fluid-structure interaction, computational fluid dynamics, inflow angle

## Abstract

The inflow angle of intracranial aneurysms (IAs) can impact the hemodynamics of IAs, therefore it is likely to contribute to IA clinical rupture risk stratification. This study aimed to assess the effect of inflow angle on the hemodynamics of IAs, as well as its potential ability to predict IA rupture risk. A novel algorithm was developed to build a series of inflow angle models on patient-specific IA models, which were reconstructed from IA 3DRA image data of eleven clinical patients. Fully coupled fluid-structure interaction (FSI) simulations were performed to quantify hemodynamic characteristics of the established IA models with various inflow angles. Hemodynamic parameters including wall shear stress (WSS), flow velocity, flow pattern, inflow zone, impingement region, pressure, and energy loss (EL) were calculated and analyzed. It was demonstrated from the analysis that a rise in the IA inflow angle is associated with the following hemodynamic changes: more direct blood flowed into the aneurysm sac, higher velocity at the upside of the aneurysm, upregulated flow velocity and WSS in the aneurysm, more complicated flow patterns, extended inflow zone, the impingement region moving upward from the neck to the apex of the aneurysm, and higher WSS and larger flow velocity at the inflow zone of the IAs. Therefore, the proposed method may be helpful in exploring the hemodynamic variations of IAs with inflow angles. The findings could be conducive to hemodynamic studies on the association between IA inflow angle and its rupture risk.

## Introduction

Intracranial aneurysms (IAs) refer to pathological dilatation of the cerebrovascular wall occurring primarily near the bifurcations of the circle of Willis (1). Their rupture and accompanying subarachnoid hemorrhage lead to high mortality and disability (2–4). However, a considerable number of IAs remain unruptured and stable during life-time follow-up (5). The management of the unruptured IAs remains challenging, and assessing the rupture risk of diagnosed IAs is critical to making clinical decisions.

Existing studies reported that the hemodynamic environment is related to the etiology and natural history of IAs. An adverse hemodynamic environment is more likely to be found in ruptured IAs (5, 6). Some hemodynamic parameters, e.g., wall shear stress (WSS), blood flow velocity, energy loss (EL), flow pattern, inflow jet, and impingement region, are tightly associated with IA rupture potential. Besides, some morphological parameters, e.g., size, aspect ratio, size ratio, height-width ratio, and inflow angle, are also linked to IA rupture. It is known that IA geometry impacts hemodynamics, and several studies attempted to delve into the impacting mechanism (7–9). Among the above-mentioned morphological parameters, the inflow angle affects blood flow into the aneurysm and is an important factor in the hemodynamic environment, the angle is even aligned with the major aneurysm growth direction (10, 11). Some researchers have investigated how the inflow angle impacts hemodynamics. Baharoglu et al. (8) analyzed 116 sidewall aneurysms. The inflow angle of the ruptured sidewall aneurysms was evidently larger than that of the unruptured sidewall aneurysms (124.9° ± 26.5° vs. 105.8° ± 18.5°, *P* = 0.0001). Moreover, they established some idealized sidewall-type saccular aneurysm models with the inflow angle ranging from 60° to 140° in a 10° increment. The results suggested that a larger aneurysm inflow angle is associated with higher peak flow velocity, larger WSS magnitude, and larger energy transmission. Lv et al. (12) harvested 108 small aneurysms and compared the morphological features between the 68 ruptured and 40 unruptured aneurysms. As revealed from their results, the two groups had a significantly different inflow angle (119° vs. 99°, *P* < 0.001), and the inflow angle appeared to be an independent risk factor in the small aneurysms. However, Tykocki et al. (13) asserted that the inflow angle had proved a weak predictor in estimating the aneurysm rupture risk. Ford et al. (14) proposed a novel parameter β, defined as an angle of the aneurysm bulb relative to the parent artery. They subsequently conducted CFD simulations on an idealized basilar tip aneurysm model with varied β (2°-30°) to analyze the relation between β and hemodynamics. These studies assessed the effect of the inflow angle on hemodynamics using idealized models, and some useful achievements were made. However, given the complexity of IAs, it is more desirable to adopt patient-specific aneurysm models to investigate how the hemodynamics vary with the inflow angle.

In this study, a tilting transformation algorithm was proposed to alter the inflow angle of an IA. Using this algorithm, we built a series of inflow angle models on each original patient-specific IA model, which was reconstructed from clinical IA 3DRA image data. Subsequently, by fully coupled fluid-structure interaction (FSI) simulation under pulsatile blood flow conditions, hemodynamic parameters, such as WSS, flow velocity, flow pattern, impingement region, inflow zone, pressure, and energy loss (EL) were calculated. The variations of the hemodynamic characteristics were analyzed to gain insights into the relationship between aneurysm inflow angle and the hemodynamics. Therefore, this study could be a useful reference for applying inflow angles to the rupture risk assessment of IAs.

## Methods

### Image Data and Original Patient-Specific IA Model

All cases in this study were collected from the Beijing Tiantan Hospital Affiliated to Capital Medical University. The inclusion criteria for this study were: (1) sidewall IAs located at the internal carotid posterior communicating artery; (2) clinical 3D-DSA images were of adequate resolution for FSI analysis. Eleven cases were randomly selected from the clinical case database of the Beijing Tiantan Hospital, and they were all unruptured. All the patients gave their written informed consent. The protocol of this study was approved by the Ethics Committee of the Beijing Tiantan Hospital Affiliated to Capital Medical University. The 3D rotational angiography (3DRA) images of these patients were acquired using a GE LCV + Digital Subtraction system (LCV; GE Medical Systems) during a 200° rotation at a rate of 8.8 frames per second. For each patient, 88 projection images were reconstructed into a 3D dataset using isotropic voxels on a dedicated GE workstation (Advantage Unix; GE Medical Systems). The acquired raw DICOM files were imported into the proprietary software Mimics 10.0 (Belgium Materialize Company). The IA geometry was extracted using a manually set image cropping threshold, and converted into a triangulated surface model. Subsequently, the surface along the normal direction of the wall was extracted to build the blood vessel wall with the software Geomagic Studio 12 (Raindrop Geomagic, Durham, USA). Next, the established model was modified as a 3D-solid volume model with the software SolidWorks 2012 (SolidWorks Corp, Concord, MA). Consequently, original patient-specific IA models for the 11 patients were established.

### Varied Inflow Angle for the IA Models

Inflow angle is the angle between two axes: one is the axis of flow in the parent vessel at the level of the aneurysm neck (referred to hereafter as the M axis); the other is the aneurysm's main axis from the center of the neck to the tip of the dome (referred to hereafter as the L axis) (8). If the parent vessel and aneurysm neck were constant, the M axis would be stable and the inflow angle would be determined only by the L axis.

**Figure 1:**
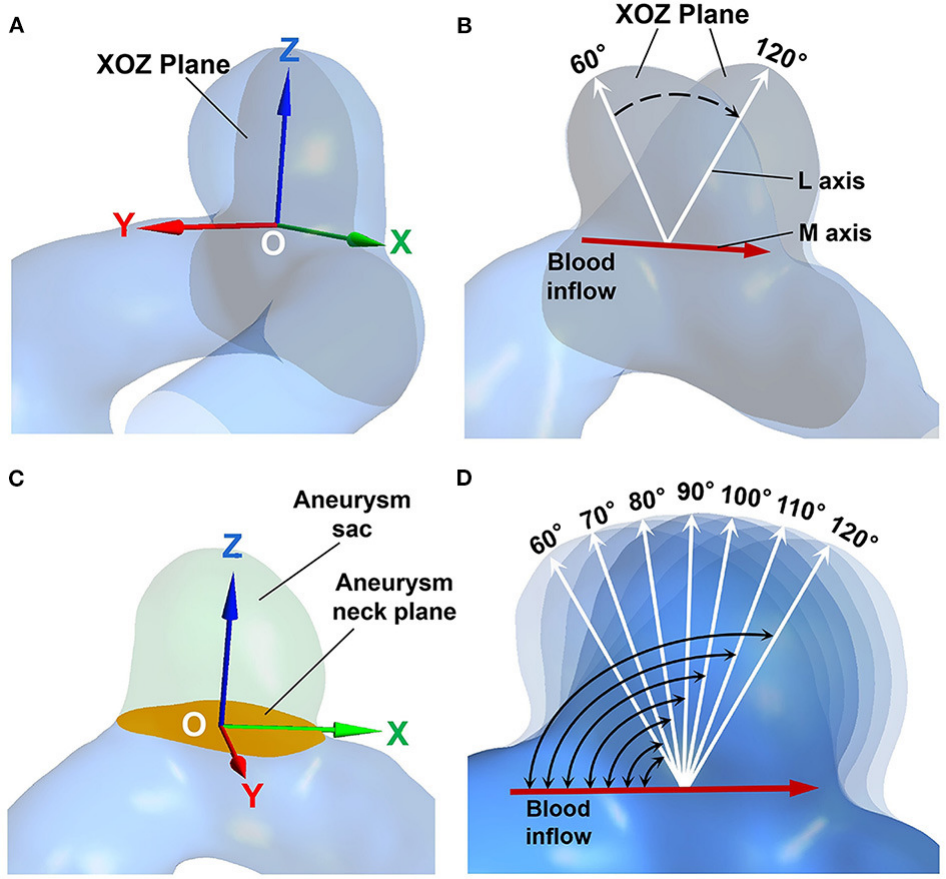
**(A)** The aneurysm geometry after rigid transformation. The center of the aneurysm neck coincides with the origin (point “O”), the gray plane indicates the XOZ plane. **(B)** The M axis (red arrow), and the L axis (white arrows) are both on the XOZ plane. **(C)** The aneurysm sac (marked in green) is separated by the aneurysm neck plane (marked in orange). **(D)** The IA models exhibiting different inflow angles.

A tilting transformation algorithm has been developed to alter the inflow angle of an IA in both positive and negative directions. In this algorithm, the IA is first rotated with a rigid transformation using the Geomagic 12 software (see Figures 1A,B), such that the center of the aneurysm neck plane coincides with the origin of the world coordinate system, the X axis is parallel to the blood flow direction in the parent vessel, and the L and M axes are both on the XOZ plane. Then the aneurysm sac is separated from the parent vessel by the aneurysm neck plane (see Figure 1C). Consequently, an IA model with a specific inflow angle is established by building new coordinates of the separated aneurysm sac, while aneurysm necks and parent vessels remain unchanged. The new coordinates of the aneurysm sac are expressed as follows:

$$\begin{array}{l} X=-\sqrt{x^{2}+z^{2}}\cdot \text{cos }\theta \\ Y=y\\ Z=\sqrt{x^{2}+z^{2}}\cdot \text{sin }\theta \end{array}$$

where *X, Y, Z*, and *x, y, z* denote the new coordinates and original coordinates, respectively. θ represents the inflow angle.

The inflow angles ranged from 60° to 120° with 10° increments for each original IA model (see Figure 1D). The range of the inflow angle was recommended by clinicians from the Beijing Tiantan Hospital.

### Numerical Simulation

In our study, the FSI method was adopted to calculate the hemodynamic parameters. The vessel was assumed to be an isotropic and hyperplastic material. The Young's modulus of the vessel, the Poisson's ratio, and the density reached 1.2 MPa, 0.45, and 1,150 kg/m^3^, respectively (15). The blood was assumed as an incompressible Newtonian fluid since slight differences between Newtonian fluid and Non-Newtonian fluid was found in blood flow simulations. The density and dynamic viscosity of blood were set to 1,050 kg/m^3^ and 0.0035 Pa·S, respectively. The average Reynold number of each original model was below 760; thus, the blood flow was considered as laminar flow. A pulsating flow, measured by the Transcranial Doppler (TCD) examination from a healthy subject, was set to be the inlet boundary condition. At the outlets, a zero pressure gradient was applied (9). A no-slip flow boundary condition was imposed on the parent vessel and aneurysm walls. The computational meshes were generated with the software ANSYS 14.0 (ANSYS Inc., Canonsburg, PA, USA). The fluid region was formed using unstructured tetrahedral elements with prismatic elements for the boundary layer. The solid region was developed with hexahedral elements. Mesh independence studies were conducted, the element size was finally set to 0.2 mm and the number of elements ranged from 8 to 10 million. The flow field and solid region analyses were conducted using ANSYS CFX and ANSYS Mechanical, respectively. The two solvers were coupled with ANSYS Workbench 14.0 (ANSYS Inc., Canonsburg, PA, USA) as a fully coupled FSI. The cardiac cycle was 0.8 s, and 2 cardiac cycles were performed in total with a constant time step of 0.01 s, and the results of the second cardiac cycle were analyzed. ANSYS CFD-Post was employed to obtain hemodynamic characteristics (e.g., mean WSS, velocity, and pressure on the aneurysm sac, flow pattern, impingement region, and EL of each established model). The method proposed by Qian et al. (16) was adopted to calculate EL. Furthermore, the correlation between the hemodynamic parameters and the inflow angle was analyzed.

## Results

The simulation results at the peak systole of the second cardiac cycle were analyzed in both quantitative and qualitative manners. The hemodynamic parameters of each established model were presented.

### Wall Shear Stress (WSS)

**Figure 2:**
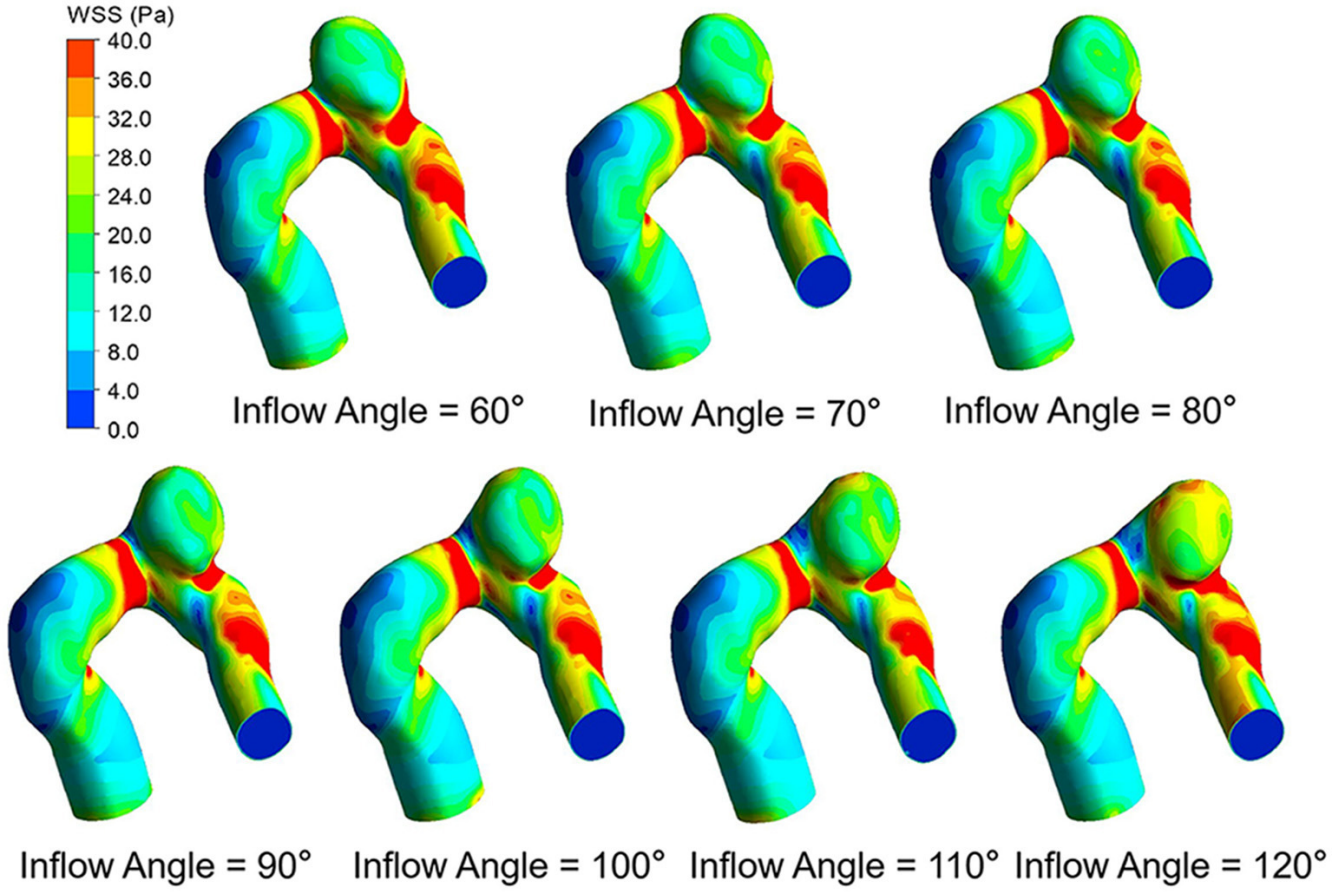
WSS distributions of seven IA models with different inflow angles at peak systole.

**Figure 3:**
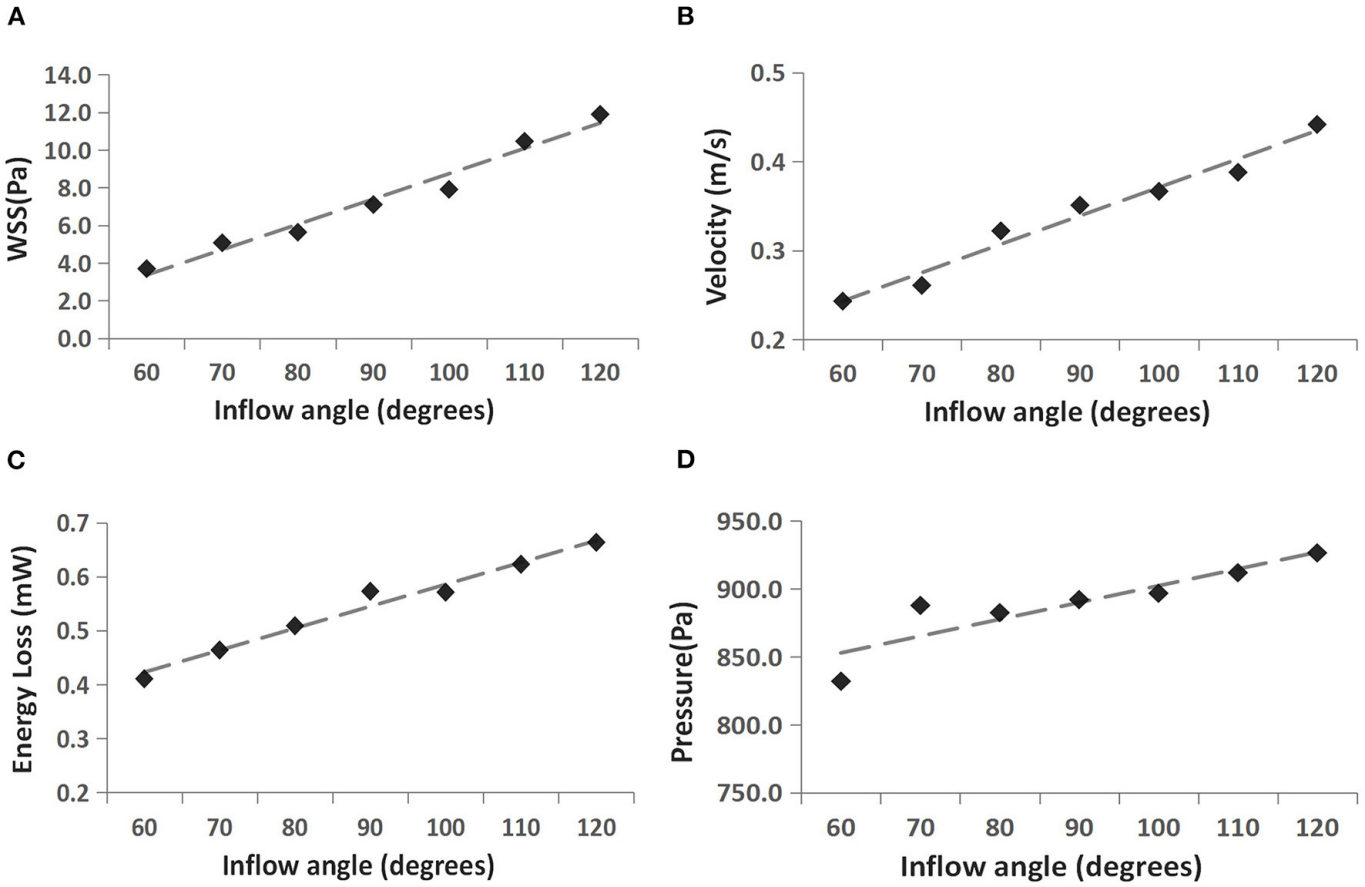
Correlation between four hemodynamic parameters and inflow angle. **(A)** WSS vs. inflow angle, **(B)** velocity vs. inflow angle, **(C)** EL vs. inflow angle, and **(D)** pressure vs. inflow angle.

The WSS distributions on the IA models with a series of inflow angle values are presented in Figure 2. The region with the maximal WSS appeared at the distal neck of the aneurysm, corresponding to the impingement region of incoming flow, and the area with the minimal WSS was located at the top of the aneurysm sac. With the increase in the inflow angle, the mean WSS on the aneurysm sac was elevated, and the low WSS area at the top of the aneurysm sac shrunk, or even disappeared. As suggested by the quantitative comparison, the mean WSS of the aneurysm sac was strongly correlated (*r* = 0.9751) to the inflow angle (Figure 3A).

### Blood Flow Characteristics

**Figure 4:**
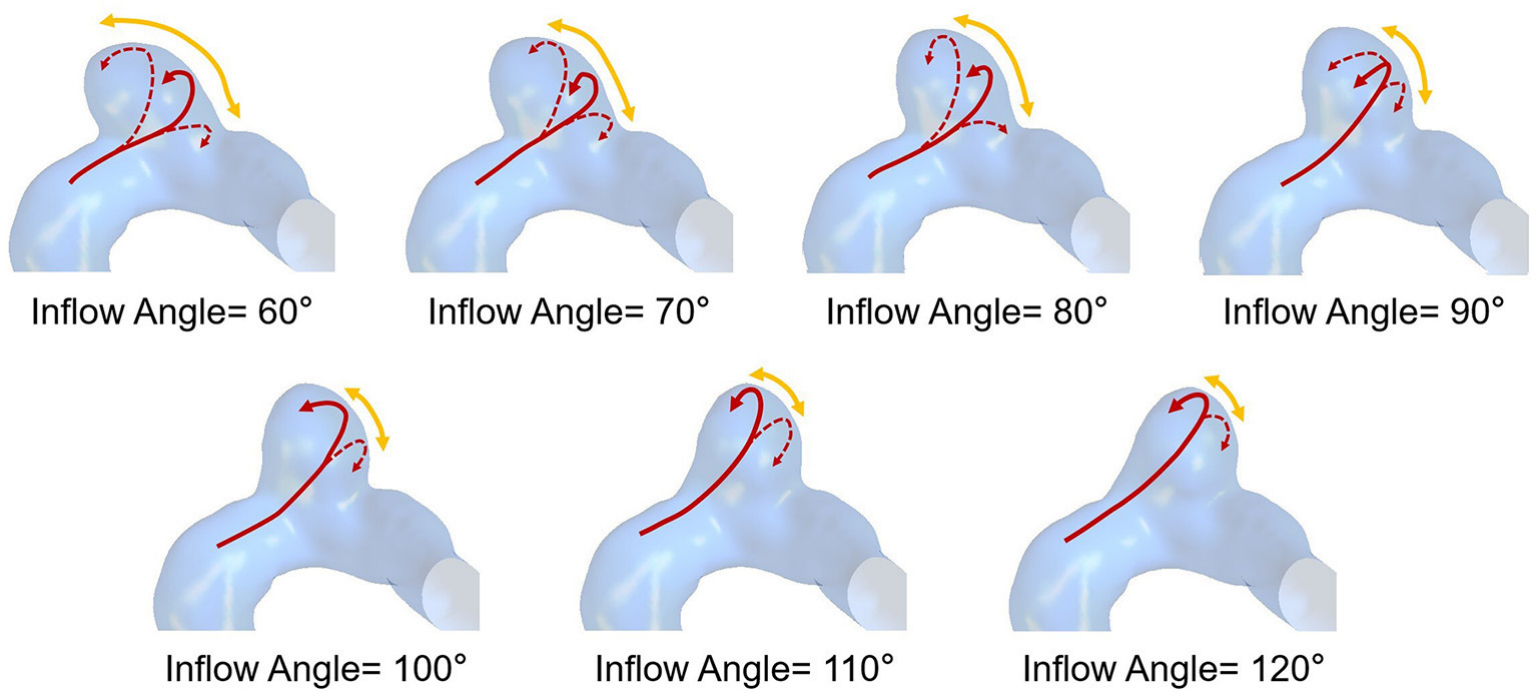
The flow pattern of seven IA models exhibiting different inflow angles at peak systole (The red solid arrows represent the direction of inflow jet, the dashed arrows represent the separate blood flow, and the yellow arrows represent the impingement region).

**Figure 5:**
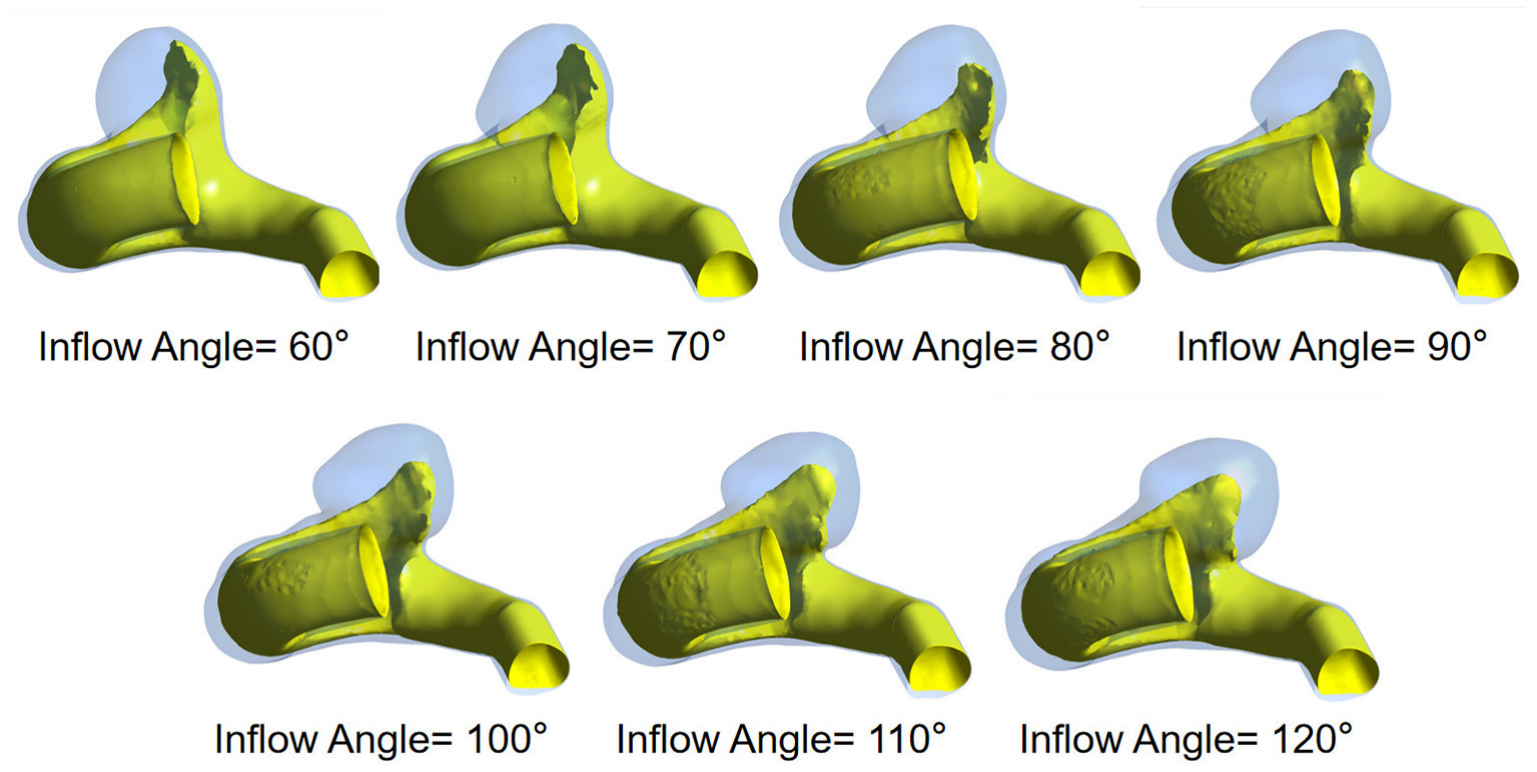
The inflow jet of seven IA models exhibiting different inflow angles at peak systole.

The flow pattern and inflow jet of the IA models with various inflow angles are presented in Figures 4, 5, respectively. The blood flew along the parent vessel, and it was split at the neck of the aneurysm. A major portion of the blood flow remained in the original direction along the parent vessel, and only a small portion entered the aneurysm sac at the distal regions of the neck. The inflow jet subsequently flew along the aneurysm wall and affected the other side. Little blood reached the apex of the aneurysm. As the inflow angle was increased, the inflow jet tended to approach the mainstream direction in the blood vessel, and more blood flow entered the aneurysm sac with higher velocity, while the impingement region moved upward. Meanwhile, the blood flow pattern became more complicated with the presence of vortices. The flow pattern and the hemodynamic parameters varied remarkably at an inflow angle of 110°. The mean velocity magnitude in the aneurysm sac showed a positive correlation (*r* = 0.8405) with the inflow angle, as illustrated in Figure 3B. EL in each model was calculated, it also correlated positively (*r* = 0.9751) with the inflow angle, as indicated in Figure 3C.

### Pressure

**Figure 6:**
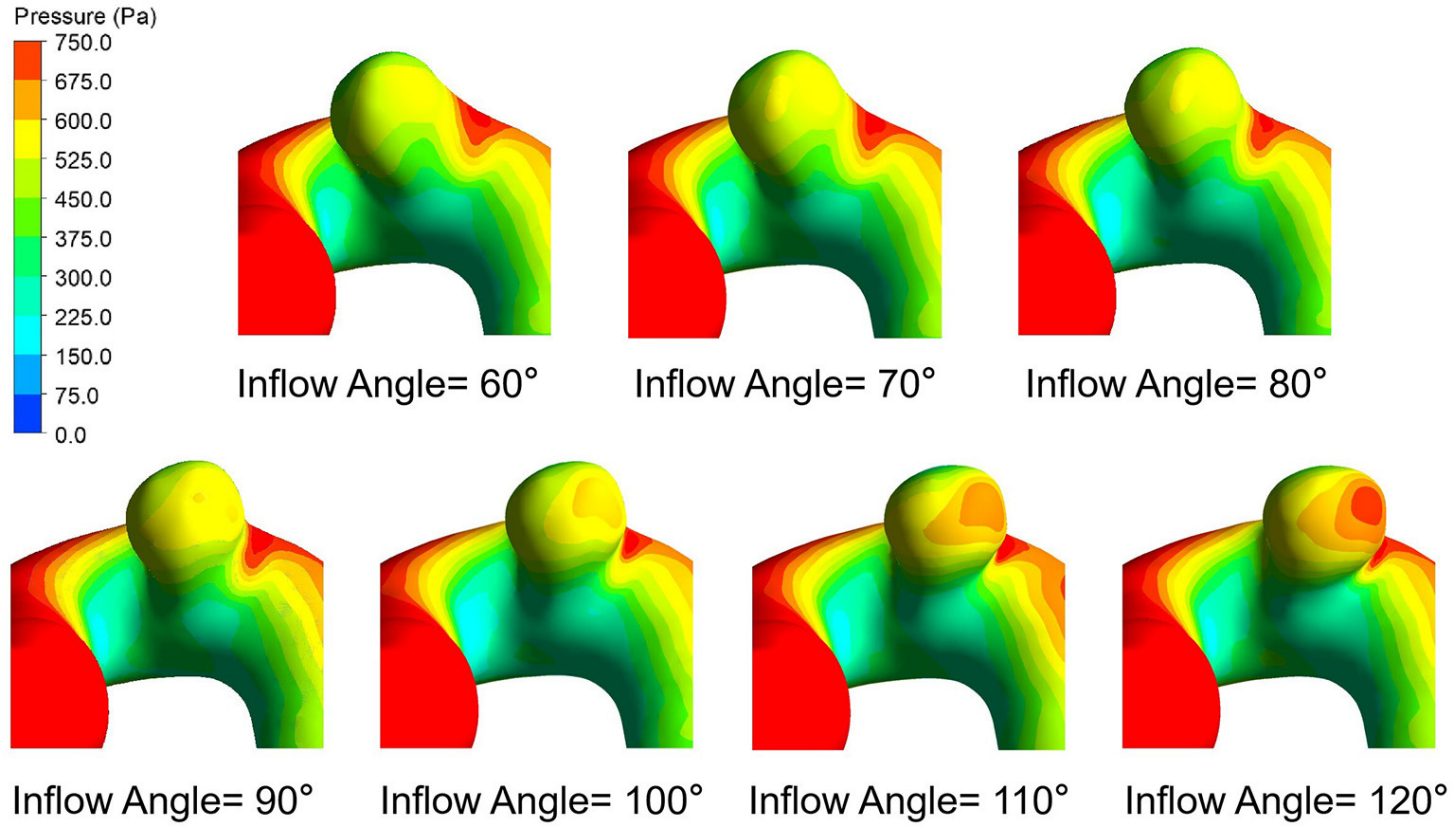
Pressure distributions of seven IA models with different inflow angles at peak systole.

Figure 6 presents the peak systolic pressure of seven IA models with different inflow angles. In each IA model, the pressure was relatively high in the impingement zone and at the top of the aneurysm sac. As the inflow angle increased, the pressure varied noticeably, and the pressure on the aneurysm dome increased. The mean pressure also exhibited a positive correlation (*r* = 0.8378) with the aneurysm inflow angle (Figure 3D).

## Discussion

In this paper, the impact of inflow angle on the hemodynamics of IAs was investigated to explore its potential ability to assess IA rupture risk. In our study, the original IA models were derived from patient 3DRA data, which reflected the patient-specific morphological features. A series of IA models with different inflow angles were established based on 11 original IA models, by applying the tilting transformation algorithm to alter the inflow angle, while the parent vessel remained unchanged. Consequently, the influence of parent vessel morphology and aneurysm neck area on hemodynamics could be excluded within the IA models. Regarding the hemodynamics of IAs, the fluid-solid coupling effect cannot be overlooked. In fact, the impingement of blood flow could cause the vessel to deform, thereby affecting the hemodynamic environment (17). Torii et al. (18) compared FSI results with those obtained from rigid arterial walls, and found that the WSS of IAs varied both qualitatively and quantitatively as impacted by the flow-wall interaction that strongly depended on the individual aneurysm shape. Given this finding, the FSI adopted in our method will be more suitable for the patient-specific hemodynamic analysis of IAs.

Although the prediction of IA rupture risk remains a challenge due to complicated mechanisms, many existing studies reported that IA rupture risks are related to a variety of hemodynamic parameters (e.g., WSS, velocity, EL, pressure, flow pattern, impingement zone, and inflow jet with high velocity). How do these hemodynamic parameters vary with inflow angle? WSS has been proven to be a critical risk factor. In the above-mentioned ideal models, WSS rises with the increase in the inflow angle, and WSS in the dome is always lower than 0.1 Pa. And the unusually low WSS is considered to be the reason for high rupture risk (8). However, by employing the proposed patient-specific IA models, we also found that WSS was elevated with the inflow angle increase, whereas all WSS was larger than 1.5 Pa, with some even rising to 13 Pa if the inflow angle >110°. Furthermore, the high WSS area in the body or at the neck increased because of the gradually invasive high-velocity blood flow impingement. The low WSS area shrunk or even disappeared, especially if the inflow angle >110°. Both high and low WSS could induce rupture risk via two different mechanistic pathways (19). It is therefore speculated that inflow angle might ultimately contribute to ruptures especially if the inflow angle >110°.

Besides WSS, other factors (e.g., velocity, EL, pressure, flow pattern, impingement zone, and inflow jet with high velocity) are also associated with IA ruptures. Our study revealed the effect of inflow angle on these hemodynamic parameters as well. As the inflow angle increases, blood flow enters the aneurysm more directly, the flow recirculation zone moves upward gradually, and the inflow jet becomes closer to the mainstream direction in the blood vessel. Consequently, the inflow jet is more concentrated with higher velocity, and the impingement zone shrinks. Besides, a relatively higher pressure forms at the IA dome. The increase in the inflow angle also causes a more complex flow pattern with more vortexes, resulting in the rise of EL. It was found that complex flow patterns, small impingement regions, and narrow inflow jet are more likely to be identified in ruptured IAs (20), and the rupture area tends to appear at the inflow zones facing the oncoming flow (21–23). Therefore, with the increase of the inflow angle, especially when it is >110°, the IA rupture risk will increase and the hemodynamic characteristics associated with higher rupture risk is expected. Besides, more rapid inflow jet impacts the aneurysm dome, and complex flow patterns form. Moreover, the impingement zone becomes smaller and more concentrated. In contrast, when the inflow angle <110°, as indicated by the simulation results, a more diffuse inflow jet flows into the aneurysm at smaller velocity, and its flow pattern becomes simpler, and the hemodynamic characteristics indicate a lower rupture risk. Based on the existing studies and our simulation results, we speculate that inflow angle may serve as an IA rupture risk factor and stratify IA rupture risk.

In the light of our simulation results, we consider that a 110° inflow angle may be the threshold linking to high rupture risk. This can be confirmed to a certain extent in existing studies. Tykocki et al. (13) suggested that the inflow angle was one of the relevant predictors, that the threshold for high rupture risk was 113.1°, and that nearly 80% of ruptured IAs exceeded the threshold. To further verify our conclusion, we also analyzed the inflow angle of ruptured and unruptured IAs in existing studies. In the study by Baharoglu et al. (24) (136 sidewall aneurysms) the inflow angles of the ruptured IAs exceeded 117.9° (range, 117.9°-132.4°); whereas the inflow angles of the unruptured group were smaller than 111.4° (range, 103.1°-111.4°). In a study by Lv et al. (12) (68 ruptured, 40 unruptured), the median value of the inflow angle of ruptured IAs reached 119°; and that of unruptured IAs was 99°. In their subsequent study [(25), 85 ruptured, 44 unruptured], the median value was 119.7° for the ruptured group, and 98.7° for the unruptured group. In addition, we also calculated the average inflow angles for the ruptured and unruptured groups based on the references (8, 13, 24). The average inflow angle for the ruptured and unruptured groups were 121.5° and 104.2°, respectively. These findings indicate that the inflow angle of ruptured IAs is more likely to exceed 110°, thus the proposed threshold in this study may be reasonable for rupture risk stratification.

In brief, a large inflow angle could induce an unfavorable hemodynamic environment with a more concentrated inflow, more complicated flow pattern, and higher WSS, inflow jet velocity, pressure, and EL. Since the inflow angle noticeably impacts the hemodynamic environment, this study suggests that inflow angle could be further studied as a potential rupture risk factor of IAs. An inflow angle larger than 110° was associated with a high rupture risk and IAs at this stage should be clinically intervened.

## Limitations

In this study, the fluid was assumed as a uniform uncompressed Newtonian fluid, and the flow was assumed to be at a steady state and in the laminar region. Although the assumptions were commonly accepted, the turbulent flow in the aneurysm sacs might be underestimated (26). The vessel wall thickness was set to be uniform, and the parameters of a normal person acquired from a Doppler ultrasound was used to set the inlet flow boundary. It is expected that the above assumptions do not undermine the accuracy of the results and affect the conclusions.

## Data Availability Statement

The datasets generated for this study are available on request to the corresponding author.

## Ethics Statement

The studies involving human participants were reviewed and approved by the Ethics Committee of Beijing Tiantan Hospital Affiliated to Capital Medical University. The patients/participants provided their written informed consent to participate in this study. Written informed consent was obtained from the individual(s) for the publication of any potentially identifiable images or data included in this article.

## Author Contributions

HL designed the research. XY contributed to the clinical data. XM and QM performed the simulation. XM and HL wrote the paper. All authors contributed to the article and approved the submitted version.

## Conflict of Interest

The authors declare that the research was conducted in the absence of any commercial or financial relationships that could be construed as a potential conflict of interest.
